# The effect of the flat-side in an engine-driven full-sequence file system on root Canal disinfection: a preliminary *ex vivo* investigation

**DOI:** 10.1186/s12903-026-07663-6

**Published:** 2026-02-02

**Authors:** Mahmoud Manaa, Soha Elhady, Jukka Matinlinna, Gianluca Plotino, Mohammed Turky

**Affiliations:** 1 Department of Endodontics, Faculty of Dentistry, Minia University, Minia, Egypt; 2 Ministry of Health and Population, Mallawy, Minia, Egypt; 3 Department of Medical Microbiology & Immunology, Faculty of Medicine, Ain Shams University, Cairo, Egypt; 4 Department of Biomaterial Science, Division of Dentistry, Manchester University, Manchester, UK; 5 Private Practice, Rome, Italy; 6 Department of Endodontics, Faculty of Dentistry, Sphinx University, Assiut,, Egypt

**Keywords:** Endodontics, *Enterococcus faecalis*, Instruments, Root canal treatment, Flat side instruments

## Abstract

**Background:**

Effective root canal disinfection is a critical component of predictable root canal treatment, yet obstacles remain in completely eradicating bacterial biofilms from the entire root canal system. Recent advances in instrument design, such as flat-side instruments, have been proposed to improve root canal disinfection and debridement. While conventional rotary instruments have been thoroughly evaluated, there is a paucity of data on the effectiveness of flat-side multi-file rotary systems in reducing the microbial burden within root canals. Therefore, this exploratory laboratory study was designed to assess the influence of the flat-side design in a full-sequence rotary file system on root canal disinfection compared to its conventional counterpart, aiming to establish initial data to guide further larger-scale research with more holistic microbial evaluations.

**Methods:**

Thirty mandibular first molars were selected according to specific criteria. After establishing an access cavity and ensuring root canal patency in all samples, the teeth underwent a cycle of autoclave sterilization. *Enterococcus faecalis* suspension was introduced into the root canals and incubated at 37 °C for 21 days. After bacterial contamination, an initial bacterial sampling (S1) was taken, and the specimens were distributed into 2 experimental groups according to the instrument design (*n* = 10 per group): flat-side full-sequence rotary files group (FS group); and its conventional prototype file system (CP group), along with positive and negative control groups (*n* = 5). At the end of the root canal preparation, another bacterial sampling (S2) was performed to determine the number of colony-forming units per mL (CFU//mL), and the results were compared with a robust ANOVA and the Games-Howell post hoc test with the significance level set at 5% and a confidence interval of 95%.

**Results:**

None of the tested endodontic instruments succeeded in completely eradicating the root canal infection. However, the flat-side instruments showed a significant reduction in bacterial load, achieving a mean value of 2.78 ± 0.11, compared to their prototype counterparts, which had a higher mean bacterial load of 3.77 ± 0.32 (p-value < 0.05).

**Conclusion:**

Instrument design can affect root canal disinfection. Flat-side instruments may be more effective in reducing the bacterial burden from root canals than their traditional counterparts. However, this conclusion was derived from a preliminary investigation conducted under specific conditions. Therefore, to enhance the reliability and generalizability of the current outcomes, it is crucial to conduct further investigations with an adequate sample size across diverse laboratory and clinical settings, as well as variations in root canal morphology, to provide a more comprehensive understanding of the impact of this design on root canal infection.

**Clinical relevance:**

The present preliminary findings suggest that while complete elimination of infection remains challenging, advancements in instrument design, such as the flat-side instruments, could enhance bacterial reduction within root canals.

**Supplementary Information:**

The online version contains supplementary material available at 10.1186/s12903-026-07663-6.

## Introduction

As endodontic disease is a biofilm-mediated infection, the ultimate goal of endodontic treatment is to eliminate the bacterial biofilm from the root canal system [1]. In an effort to successfully execute an effective endodontic treatment based on a solid scientific foundation, a thorough knowledge of the microbiologic aspects of root canal infections is essential [2]. Disinfection of the root canal system is quite difficult due to the intricate and variable root canal anatomy, along with the complex multi-species microbial communities termed biofilms [3]. Moreover, the bacterial aggregations in the biofilm are embedded in a self-produced polymeric extracellular matrix, which provides a pivotal role in the ecology, morphology, survival, and resistance of the microbial biofilm community [4]. Research has linked the effective removal of biofilm to a chemo-mechanical process, which involves the use of mechanical instruments and chemical disinfectants such as root canal irrigants and/or intracanal medicaments [3].

While chemical disinfecting agents have been reported to reduce the intra-radicular bacterial load significantly [5–7], mechanical instrumentation accounts for more than 95% of the bacterial reduction. Research has highlighted that root canal instrumentation can provide the most effective method to detach the bacterial biofilms from the canal walls [8–11]. This method can surpass the disinfecting chemical agents, which struggle to penetrate the biofilm structure due to the extracellular matrix that represents the impregnable fortress for the bacterial agglomeration within the biofilm [8–11].

To date, a substantial body of literature has revealed that there are no significant differences observed between manual and automated mechanical instrumentation in terms of root canal disinfection [12–15]. However, automated nickel-titanium (NiTi) systems offer several merits over manual techniques, including shorter shaping duration [16, 17], more centered preparations with less transportation, and a lower incidence of canal aberrations, particularly in curved canals [18–20], less debris extrusion [21], and more favorable clinical outcomes [22]. Given that, several rotary systems have evolved that differ in multiple aspects, including the sequence, kinematics, metallurgy, manufacturing process, surface treatment, and design, aiming to improve the quality of root canal preparation [11]. It has been demonstrated that instrument design can affect root canal disinfection [11].

The flat-side rotary instruments are one of the latest modifications in instrument design that have sparked much interest in the endodontic community. The first emergence of the flat-side design was in 2015 with the Tango-Endo system made of austenitic Niti alloy (Essential Dental Systems, South Hackensack, USA). Since then, numerous heat-treated Niti rotary files with a flat-side design have emerged such as SafeSiders HF (Essential Dental Systems, South Hackensack, USA), Fury L60 (Mighty Medico, Changzhou, Jiangsu Province, China), M3 L Platinum (United Dental Group, China), AF F-One Blue (Fanta Dental Materials Co. Ltd., Shanghai, China) and Platinum V.EU (United Dental Group, China).

All manufacturers have consistently asserted that this distinctive design offers multiple advantages. Firstly, it could provide superior flexibility and resistance to cyclic fatigue due to the reduced metal mass achieved by the flat cross-section. Secondly, it may enhance cleaning capabilities by facilitating the removal of debris from the flutes to the relieving areas along the flat side. Thirdly, the decreased engagement with the canal walls is expected to prolong the lifespan of the instruments. Lastly, the design may support effective chemical cleaning and disinfection by allowing the storage of irrigating solutions within the root canals during instrumentation procedures. All these theoretical privileges have been investigated in scientific research with controversial results. Some studies proved the superiority of flat-side instruments over the conventional design in some aspects, such as cyclic fatigue resistance, shaping and cleaning abilities, and cutting ability [23–26]. Contrarily, other investigations showed their inferior mechanical performance regarding flexural fatigue, torsional strength, angle of rotation, buckling strength, and flexibility without notable differences in terms of preparation time, percentages of untouched walls, debris removal, and root canal volume between the flat-side files and their counterparts [26–31].

Despite the considerable disparity witnessed in the scientific literature concerning flat-side rotary instruments, the disinfecting ability of flat-side instruments has not been sufficiently and adequately investigated. Only one study evaluated the ability of the flat-side instrument to eliminate intra-radicular biofilm using a single-file system with a small apical preparation size (25/0.06) in single-rooted mandibular premolars with single straight oval root canals and found inferior disinfecting capabilities [32].

The ongoing research remains inconclusive in resolving the debate regarding the efficiency of single-file versus multiple-file systems in the disinfection of root canals [33–37]. However, multi-file systems allow for multiple instrument insertions into root canals with different angles of insertion, resulting in more interactions with larger surface areas of the root canal walls, thereby enhancing the mechanical action of the file in biofilm detachment from the canal walls [37]. Furthermore, introducing multiple files into the root canals is generally linked to increased volumes and prolonged exposure times to irrigants, thereby improving bacterial reduction within the root canals [37, 38]. Conversely, a single-file system is associated with fewer insertions and limited volumes and time of root canal irrigation [37]. Considering the essential role mechanical instruments play in root canal disinfection and to fill a knowledge gap, the present preliminary study sought to evaluate the influence of full-sequence flat-side instruments on intraradicular bacterial reduction in an attempt to provide baseline data that could inform future studies with larger sample sizes and more enhanced bacterial assessments. Therefore, the present ex vivo investigation aimed to evaluate the impact of the flat-side design in a multi-file rotary system on root canal disinfection compared to its conventional counterpart.

The null hypothesis would be that there is no significant difference between flat-side multi-file rotary systems and their classic counterparts regarding root canal disinfection.

## Materials and methods

### Ethical regulation

The present study was carefully conducted in adherence to the ethical guidelines established by the Declaration of Helsinki, which emphasizes the importance of protecting the rights and welfare of participants in medical research. Prior to the initiation of the study, ethical approval was secured from the Research Ethics Committee at the Faculty of Dentistry, Minia University, Egypt, under Committee No. 108 and Registration No. 928. This approval process involved a thorough review of our research protocol to ensure compliance with all relevant guidelines and regulations pertaining to research on human subjects.

In addition, the present investigation prioritized transparency and voluntary participation by obtaining written informed consent from all individuals who participated in the study. This consent process included providing potential participants with comprehensive information regarding the study’s objectives, procedures, potential risks, and benefits, allowing them to make informed decisions about their involvement. All methods and practices throughout the study were diligently implemented to uphold the highest ethical standards and to respect the dignity of all participants.

### Sample size calculation

Despite being a preliminary study, the present study strived to introduce valid initial observations. As a result, the sample size was estimated based on a pilot study. A pilot study was conducted involving the analysis of five teeth per group. The data collected from this study were then subjected to a power analysis using G*Power software to determine the optimal sample size necessary for subsequent research phases. The power analysis was configured with a significance level (alpha, α) set at 0.05, indicating the threshold for statistical significance. Additionally, a beta (β) level of 0.95 was employed, corresponding to a desired statistical power of 95%. This high power level is essential to ensure a strong probability of detecting a true effect, should one exist. Furthermore, an effect size (f) of 1.18 was utilized for the one-way ANOVA test, reflecting a substantial effect expected from the intervention or treatment under investigation. Based on these parameters, the analysis revealed that a total sample size of 20 teeth would be required, allocating five teeth to each group.

### Sample selection

A total of 30 freshly extracted intact human mandibular first molars, each featuring fully developed two roots, were meticulously collected from patients aged 20–40 years at the outpatient clinic, Faculty of Dentistry, Minia University, Minia, Egypt. These teeth were extracted specifically for periodontal reasons.

To ensure the anatomical consistency of the selected teeth, a comprehensive anatomical matching process was conducted. This evaluation focused on tooth morphology, dimensions, and the morphology and volume of the pulp space, utilizing cone-beam computed tomography. The selected molars demonstrated equivalent angles of root and root canal curvatures, which were assessed using Schneider’s method [39], a standardized technique for analyzing canal curvatures. Additionally, the radius of the curvatures was consistently less than 5 mm, and all teeth exhibited an identical root canal configuration, specifically conforming to Vertucci type IV classification [40] within the mesial root. To facilitate adjusting the working length across the different samples, teeth where the canal terminus coincided with the root apices were selected.

The matching process was carried out by two experienced and calibrated examiners, each of whom independently evaluated all specimens. Prior to the commencement of data collection, a comprehensive calibration session was conducted to minimize interpretative variability and to enhance the methodological rigor of our study. During this calibration, the examiners reviewed and discussed specific evaluation criteria, which facilitated a shared understanding of the assessment framework. This approach resulted in a high inter-observer agreement rate of 90%, indicating a high level of consistency in their assessments.

In instances where discrepancies arose between the examiners’ evaluations, they collaboratively engaged in a consensus discussion to resolve differences. This collaborative approach ensured that the final evaluations were consistent, reinforcing the reliability of our assessment methods. Notably, the level of agreement achieved in the present study surpasses the accepted and reliable levels that were reported in previous research, which indicated inter-observer agreement rates ranging from 82% to 86% [41]. This additional evidence further supports the dependability and integrity of our assessment approach, providing confidence in the findings presented.

Teeth that presented with caries, prior restorations, previous root canal treatments, cracks, fractures, internal or external root resorption, and pulp calcifications were systematically excluded. This exclusion was vital to maintain the integrity of the sample, and any disqualified teeth were promptly replaced with others that fulfilled the stringent inclusion criteria established for this research.

All selected teeth underwent a thorough cleaning process to remove any soft or hard deposits. Following this, they were disinfected using a 5.25% sodium hypochlorite solution (NaOCl) with a disinfection duration of 30 min to ensure adequate sterility. To preserve the integrity of the samples until the time of use, the teeth were subsequently stored in a 0.1% thymol solution at 4 °C for less than one month.

### Sample preparation

#### Access cavity preparation and creating a glide path for bacterial inoculation

Standardized traditional access cavities were prepared following the common guidelines for access cavity preparation to achieve straight-line access to the canal orifices and/or the initial canal curvatures [42]. Aligning with those guidelines and to ensure aseptic and standardized procedures, the access cavity was initially prepared using a sterile round diamond bur # 801 − 014 while the axial walls were refined using a tapered carbide fissure bur #H33L, creating slightly divergent cavity walls to improve the visibility and accessibility. All the burs used for the access cavity preparations were autoclaved at 121 °C for 15 min prior to use and adapted on a sterile high-speed handpiece with sterile distilled water spray to ensure the sterility of the procedures. The prepared access cavity was soaked in 5.25% NaOCl for 5 min. Subsequently, the patency of the mesial root canals (mesiobuccal and mesiolingual) was checked using a sterile stainless-steel K-file ISO size 10, followed by 2 mL of 5.25% NaOCl irrigation for 20 s. Otherwise, non-negotiable canals or those with initial apical diameters larger than size 15 were discarded and replaced with the same strict criteria previously described.

The occlusal surfaces were reduced to standardize the teeth’s length at 19 mm. Afterward, the working length was determined by inserting a sterile stainless-steel K-file ISO size 10 till it was observed through the apical foramen. Then, 0.5 mm was subtracted from this measurement, and a fixed reference point was determined for each canal in all samples. All mesial root canals were enlarged up to a sterile stainless-steel K-file ISO size 20 under irrigation with 5 mL of 5.25% NaOCl for 1 min, which was then inactivated with 10% sodium thiosulfate. While this initial root canal preparation aimed to provide adequate room for efficient bacterial inoculation along the entire canal length alongside consistent bacterial distribution across the different samples, smear layer formation is a potential concern that may impede proper biofilm adherence to the canal walls. To mitigate this issue, 5 mL of 17% ethylenediaminetetraacetic acid solution (EDTA) was administered for 1 min, followed by a final flush of saline with the same exposure time and volumes to eliminate the persistent effect of EDTA on the canal walls.

All the root surfaces were covered with nail varnish to prevent the possibility of bacterial leakage. The coronal cavity was hermetically sealed with a temporary restoration (Cavit G). To rule out the presence of any bacterial infection, all teeth were sterilized in an autoclave for 15 min at 121 °C after being placed in 20 mL Falcon tubes containing distilled water. To verify adequate sterilization, each root canal was flushed with 1 mL of saline solution, which was evenly dispersed throughout the full root canal length using a sterile stainless-steel K-file ISO size 15. Next, culture was taken after circumferential filing of the root canal walls using a sterile stainless-steel H-file ISO size 15, followed by sequential insertion of three paper points ISO size 15 inside the root canal, each for 1 min. Subsequently, the samples were plated onto brain-heart infusion agar plates and incubated at 37 °C for 21 days. For easier identification and handling, the teeth were mounted vertically in acrylic resin and placed in Eppendorf tubes.

#### Bacterial inoculation

The clinical isolate of *Enterococcus faecalis* (*E. faecalis*), designated with the American Type Culture Collection (ATCC) number 29,212, was cultivated in brain-heart infusion (BHI) broth (from Oxoid, CM225) for 24 h at an optimal incubation temperature of 37 °C. Following this incubation, the bacterial concentration was adjusted to exceed 10^8^ colony-forming units (CFU)/mL, ensuring a robust suspension ideal for experimental use. The concentration of the bacterial suspension was meticulously adjusted by measuring the optical density at a wavelength of 600 nm (OD₆₀₀) using a UV–Vis spectrophotometer. Prior to measurements, the spectrophotometer was calibrated to ensure accuracy, and a blank control was utilized to eliminate any background interference. The suspension was standardized according to a pre-established correlation between OD₆₀₀ readings and the viable counts of *E faecalis*, enabling reliable quantification of the bacterial concentration. This correlation was derived from serial dilutions of *E. faecalis*, which were plated on appropriate agar media, allowing for the determination of cell viability in relation to optical density. This standardized approach ensured that the readings accurately reflected the concentration of live bacteria in the suspension, which is critical for subsequent experimental protocols.

To prepare the next phase of experimentation, 1 mL of the prepared *E. faecalis* broth was transferred into a fresh BHI medium. Utilizing sterile micropipettes, each root canal received an inoculation of 10 µL of the *E. faecalis* suspension. The inoculum was then carefully dispersed throughout the entire canal length using a sterile K-file ISO size 15, ensuring even distribution of the bacteria within the canal. After the inoculation, the canal orifice was sealed with sterile cotton.

Subsequently, the specimens were incubated in *E. faecalis* suspension at a concentration exceeding 108 CFU/mL for a continuous duration of 21 days at 37 °C. To sustain the bacterial culture, fresh *E. faecalis* suspension was refreshed every three days [43, 44]. The present study made a deliberate choice to renew the *E. faecalis* suspension rather than merely replacing the culture medium. This decision was grounded in the goal of maintaining optimal bacterial viability, metabolic activity, and consistent infection conditions throughout the 21-day incubation period. The rationale for this approach can be understood through the following key factors:

##### Maintaining bacterial viabiliy and density

By renewing the bacterial suspension, we ensure that the concentration of *E. faecalis* remains consistent. This is critical because factors such as cell death, nutrient depletion, and pH alterations due to metabolic byproducts can occur over time. Replacing the medium without reintroducing fresh bacterial cells can significantly decrease bacterial density. This could lead to suboptimal levels of infection, as a higher bacterial load is essential for mimicking clinical conditions accurately.

##### Nutrient replenishment and waste removal

Introducing a fresh suspension serves a dual purpose: it provides the essential nutrients needed for sustained bacterial growth and biofilm formation while simultaneously diluting and removing accumulated waste products. Waste accumulation can create an unfavorable microenvironment that inhibits bacterial growth, particularly in terms of lowering pH levels. By implementing a strategy of suspension renewal, the present study ensured that the bacteria have access to a rich nutrient environment, which is vital for their metabolic processes and overall growth.

##### Ensuring consistent infectivity

*E. faecalis* is renowned for its ability to form resilient biofilms, especially within root canal models, where biofilm formation is a critical factor influencing treatment outcomes. Regular renewal of the suspension supports the continuous development of biofilms by introducing metabolically active cells that can contribute to their growth and maintenance. This practice enhances the clinical relevance of the infection model, as it allows for better simulation of the dynamics of a real-world infection scenario over the duration of the study.

In summary, the renewal of the *E. faecalis* suspension—rather than relying solely on the exchange of culture medium—was deemed essential for maintaining a high-density, metabolically active bacterial population throughout the experiment. This approach ensures reproducible and clinically representative infection conditions, ultimately enhancing the reliability and validity of our study outcomes.

For monitoring purposes, two distinct bacterial samplings were conducted for each specimen: the first sampling occurred before root canal preparation, and the second sampling was performed post-preparation, allowing for a comprehensive comparison of bacterial viability throughout the experimental process [36].

#### Bacterial sampling before root canal preparation (S1)

This procedure was meticulously designed to achieve a consistent and homogeneous distribution of bacteria across the various study samples, thereby enhancing the reliability of the results. Initially, root canals were irrigated using a sterile saline solution. This saline was carefully distributed along the entire length of each root canal utilizing a sterile K-file ISO size 15, ensuring thorough irrigation of the canal.

Following the initial irrigation, a sterile stainless-steel H-file, also ISO size 15, was employed. The H-file was manipulated in a controlled pumping motion—consisting of an in-and-out technique—against the walls of the root canal, extending to the predetermined working length. This method aimed to further disrupt any remaining debris and bacteria adhered to the canal walls.

Subsequently, three sterile paper points ISO size 20 were sequentially inserted into each root canal, with each point left in place for 1 min. This step allowed for the optimal absorption of residual microorganisms, as well as any remaining irrigation fluid. To remove the paper points efficiently and maintain sterility, sterile tweezers were utilized. The withdrawn points were then promptly placed into sterile plastic tubes that contained 1 mL of physiological saline solution for subsequent analysis.

Once the paper points were secured in the tubes, they were vortexed for 30 s to ensure the effective release of the bacteria into the saline. Then, 20 µL aliquots from the saline solution were taken for plating on BHI agar plates after performing a series of ten-fold dilutions in sterile saline. This dilution process was critical to quantify bacteria accurately without overloading the agar plates. The plates were then incubated at 37 °C for 48 h, allowing for optimal bacterial growth and subsequent colony formation for analysis [10].

### Sample classification

The sampling distribution employed a stratified approach, wherein specimens were meticulously paired based on their dimensions, anatomical features, and ages to ensure a comprehensive analysis. Teeth exhibiting comparable dimensions, anatomical characteristics, and age ranges were systematically classified into four distinct groups. This classification aimed to create a robust framework for comparison, with each group consisting of four teeth that were anatomically and dimensionally matched while also reflecting similar developmental stages. This careful categorization was deliberately designed to minimize potential bias arising from anatomical variations, thereby enhancing the reliability and validity of the research findings related to dental characteristics and their implications.

Following contamination of root canals with *E. faecalis*, the teeth were distributed into two experimental groups (*n* = 10) based on the instrument design and two control groups (*n* = 5) as follows:

- *Experimental group 1 - root canal preparation with flat-side full-sequence rotary files (FS group)*:

In this group, root canals were instrumented in a crown-down technique, following the contemporary guidelines for root canal preparation [45], using AF F One full-sequence flat-side rotary files (LOT: 112221114005) mounted on an electrical torque-controlled endodontic motor (TriAuto mini). The torque and speed were adjusted according to the manufacturer’s instructions. This multi-file system consists of sizes 17/0.12, 20/0.04, 25/0.04, 30/0.04, and 35/0.04 used in a continuous rotation motion. All the files were sterilized in an autoclave at 121 °C for 20 min, and only one set was utilized for each canal. File size 17/0.12 was used for coronal flaring, while the instrument sizes 20/0.04, 25/0.04, 30/0.04, and 35/0.04 were used sequentially up to the pre-established working length in a brushing motion with a light apical pressure up to the apical one-third to facilitate the file’s progression toward the apex with the least resistance. A pecking motion was employed in the apical part to maintain the cross-section of the root canal anatomy. Following each 1–2 mm incremental apical progression, the instrument was withdrawn from the canal, and the accumulated debris between flutes was removed using a sterile sponge to improve the file’s cutting ability and minimize the possible stresses on both files and dentin. After each mechanical file insertion, the patency of the root canals was secured using a sterile stainless-steel K-file ISO size 10, followed by root canal irrigation with 2 mL of 5.25% NaOCl for 20 s using a 30-gauge side-vented closed-end needle adapted on a 3 mL plastic syringe and inserted 1 mm short of the working length with an up-down movement while the irrigant was injected. At the end of the root canal instrumentation, a final rinse of alternate use of 10 mL of NaOCl for 2 min, followed by 10 mL of 17% EDTA for 2 min, was employed, with intermediate irrigation using the same volumes of saline solution for 2 min. A final flush with saline solution was carried out to eliminate any residues of the irrigating solutions.

Each root canal received a total of 20 mL of 5.25% NaOCl for 220 s, 10 mL of 17% EDTA for 120 s, and 20 mL of saline irrigation for 240 s. Moreover, the average instrumentation time was 10 min per canal.

- *Experimental group 2 – root canal preparation with the conventional prototype file system (the conventional AF Blue file system) (CP group)*:

In this group (*n* = 10), root canal procedures using the conventional AF Blue file system (LOT: 112221101001) were performed in the same manner as previously mentioned in the FS group. Like the AF F One flat-side rotary system, the AF Blue file system is also a multi-file system with the same design except for the flat side, comprising the same instrument sizes and tapers and using the same kinematics (a continuous rotation motion).

- *Control group 1 –* positive control group (PC):

In this group (*n* = 5), the root canals were infected with *E. faecalis*, but were not prepared to monitor the bacterial viability and growth (load) throughout the experimental procedures.

- *Control group 2 – negative* control group (NC):

In this group (*n* = 5), the root canals were prepared but not infected to assess the sterility of the procedures and establish a baseline for comparison.

It is worth mentioning that 10% sodium thiosulfate was used to neutralize the actions of NaOCl.

### Estimation of bacterial count

A second bacterial sampling was performed after root canal preparation (S2). The root canals were irrigated with 2 mL of saline solution, which was evenly disseminated using a sterile stainless-steel K-file ISO size 15. A small sterile stainless-steel H-file (ISO size 15) was used to scrape the canal walls circumferentially up to the working length. Subsequently, three sterile paper points, size 35, were inserted successively for bacterial culture, each for 1 min, using the same approach as before the chemo-mechanical preparation. The colonies on every plate were counted, and the number of colony-forming units per 1 mL was determined and expressed as CFU/mL [10].

The manufacturer’s information for all devices, instruments, and materials used in the present study is listed in Table 1.

**Table 1:** The manufacturer’s information for devices, instruments, and materials utilized in the present study

Devices	Manufacturer’s information
G*Power software	Version 3.1.9.7, Heinrich Heine University, Düsseldorf, Germany
Cone-beam computed tomography	Papaya 3D Plus, Genoray, Gyeonggi-do, Korea
UV–Vis spectrophotometer	Thermo Fisher Scientific, Waltham, MA, USA.
TriAuto mini	J. Morita MFG, CORP, Japan
Instruments	Manufacturer’s information
A round diamond bur	Komet Den Komet Dental, Braseler GmbH & Co. KG, Lemgo, Germany
A tapered carbide fissure bur	Komet H33L, Komet Den Komet Dental, Braseler GmbH & Co. KG, Lemgo, Germany
K-file ISO sizes 10, 15, and 20	Dentsply, Maillefer, Ballaigues, Switzerland
H-file ISO size 15	Dentsply, Maillefer, Ballaigues, Switzerland
AF F One full-sequence flat-side rotary files (LOT: 112221114005)	Fanta Dental Materials Co., Ltd., Shanghai, China
The conventional AF Blue file system (LOT: 112221101001)	Fanta Dental Materials Co., Ltd., Shanghai, China
A 30-gauge side-vented closed-end needle	Fanta Dental Materials Co., Ltd., Shanghai, China
Materials	Manufacturer’s information
5.25% Sodium hypochlorite solution (NaOCl)	Omez, Phar Omez, Pharaonic Pharmaceuticals, Egypt
0.1% thymol solution	Formula e Acao, São Paulo, SP, Brazil
17% ethylenediaminetetraacetic acid solution (EDTA)	Prevest DenPro Limited, Jammu & Kashmir, India
A temporary restoration (Cavit G)	3 M ESPE, Neuss, Germany
Paper points ISO size 15 and 35	Dentsply, Maillefer, Ballaigues, Switzerland

### Variable control

In an important endeavor to achieve valid and reliable outcomes and ensure the reproducibility of the methodology, the present laboratory investigation was conducted under controlled conditions. Therefore, the following strategies were meticulously executed:

#### Sample standardization

The same tooth type (intact human mature mandibular first molar) was selected, and similar samples in terms of dimensions, anatomy, and age were distributed equally to the distinct study groups. Prior to commencement of experimental procedures, all samples were cleaned, disinfected, and stored in the same solutions for the same storage time and temperature. Subsequently, the samples underwent the same pre-instrumentation enlargement before being submitted to standardized sterilization conditions to allow consistent bacterial inoculation.

#### Operator experience and skills

All the root canal procedures were performed by a specialist with 10 years of experience in endodontics (M.M.) in a single setting. The implementation of operator blinding was not feasible in this context due to the distinct visual characteristics of flat side instruments compared to their conventional counterparts. On the other hand, the evaluation process was meticulously carried out by a dedicated examiner who was intentionally blinded to the specific study groups (S.E.). This careful approach aimed to eliminate any biases and ensure that each sample was assessed fairly and without preconceived notions. By maintaining this level of objectivity, the integrity of the results was upheld, allowing for a more accurate understanding of the findings and their implications.

The statistical analysis was conducted by a highly experienced specialist who brings three decades of expertise in data analysis. Importantly, this professional had no involvement in the current investigation, ensuring an impartial assessment of the study’s findings. By leveraging advanced analytical techniques and methodologies, the expert was able to assess the data, providing a rigorous and unbiased interpretation that enhances the credibility of the research outcomes.

#### Standardization of chemical agents and other root canal procedures to isolate the effect of instrument design

A precise standardization of the concentrations of the employed chemical solutions using the iodometric titration method was conducted. This analytical approach is critical for accurately measuring the chemical composition and, hence, achieving precise quantification of the concentration level of the different solutions, either the irrigants or their neutralizing agents.

In addition to standardizing the concentrations of the solutions, the total volumes and irrigation duration were consistently calibrated. Moreover, the penetration depth of the irrigation needles was adjusted to be 1 mm shorter than the working length across all groups. This was achieved by placing the silicone stopper at a fixed reference point.

Furthermore, the instrumentation apical size (0.35 mm), taper (0.04), and time, along with the number of in-and-out movements of the files, were standardized. Additionally, the instruments being compared share similar metallurgy and kinematics. This approach ensured that each group was subjected to consistent operational parameters, allowing for a more precise comparison of the study outcomes.

#### Standardization of biofilm maturation (biofilm age)

The biofilms in all samples were incubated under identical specific environmental conditions (37 °C, microaerophilic conditions, BHI, and pH ranged from 7 to 7.5) for the same duration (21 days) to ensure a uniform biofilm maturation, allowing for a more effective evaluation of the disinfection process across the different study groups.

#### Environmental conditions

Heating systems and air conditioning were employed to provide a temperature-controlled room with a stable temperature of 37 °C. In addition, humidifiers and dehumidifiers were utilized to ensure constant humidity levels.

Ambient conditions were continuously checked throughout the experiment using digital thermometers and hygrometers to ensure stability and detect any fluctuations.

To minimize variations, all samples were evaluated on the same day. Moreover, the samples, devices, instruments, and materials were kept in the same room to establish thermal equilibrium before testing.

By maintaining uniformity in these key aspects, the present experiment aimed to mitigate the risk of bias, thereby enhancing the reliability of the findings.

### Statistical analysis

The initial assessment of the numerical data was conducted to determine whether it adhered to a normal distribution, employing the Shapiro-Wilk test. The analysis revealed that the bacterial count data did not follow a normal distribution, exhibiting a significant positive skewness. In order to rectify this skewness and meet the assumptions necessary for subsequent statistical analyses, the data underwent a log transformation.

Furthermore, Levene’s test was conducted to assess the homogeneity of variances across groups; the results indicated significant unequal variances among the samples. Consequently, to accommodate these violations of assumptions, a robust analysis of variance (ANOVA) was performed, supplemented by the Games-Howell post hoc test to explore differences between group means. The *t*-test was utilized to verify the uniformity of bacterial distributions between the two experimental groups prior to the introduction of instrumentation. This statistical analysis ensured that any observed differences in bacterial counts could be attributed solely to the effects of the instrument designs rather than variations in baseline bacterial populations. Additionally, the *t*-test was employed to evaluate the effectiveness of each instrument design in reducing bacterial counts. By comparing the mean bacterial counts before and after instrumentation within each group, clear conclusions were drawn about the efficacy of the different designs in mitigating bacterial presence.

The outcomes of this analysis are reported as mean values accompanied by their respective standard deviations, with a significance threshold set at 5%. All statistical computations were executed using R Statistics (version 4.1.3 for Windows, Bell Laboratories, Murray Hill, NJ, USA). This methodological approach ensured a rigorous examination of the data, allowing for reliable interpretations of the bacterial counts.

The effect size was calculated at 1.18, indicating a clinically significant difference between the intervention groups under evaluation. This substantial effect size underscores the potential efficacy of the tested instruments in effectively reducing bacterial load in root canal treatments, thereby enhancing their applicability in clinical endodontics.

Additionally, the study provided an in-depth analysis of the 95% confidence interval (CI) related to the effect size, which is crucial for assessing the statistical reliability and precision of the findings. A narrow CI suggests a high degree of certainty about the disinfecting effectiveness of the evaluated instruments, indicating that the results are both consistent and reproducible. Conversely, a wider CI could reflect variability within the dataset, highlighting the need for further investigation into the factors that may underlie the observed discrepancies.

The combination of a notable effect size and robust confidence intervals not only reinforces the strength of the evidence but also emphasizes the potential clinical implications of these findings. This suggests that the integration of the evaluated instrument design in practice could improve treatment outcomes for patients undergoing root canal treatment.

## Results

**Fig. 1:**
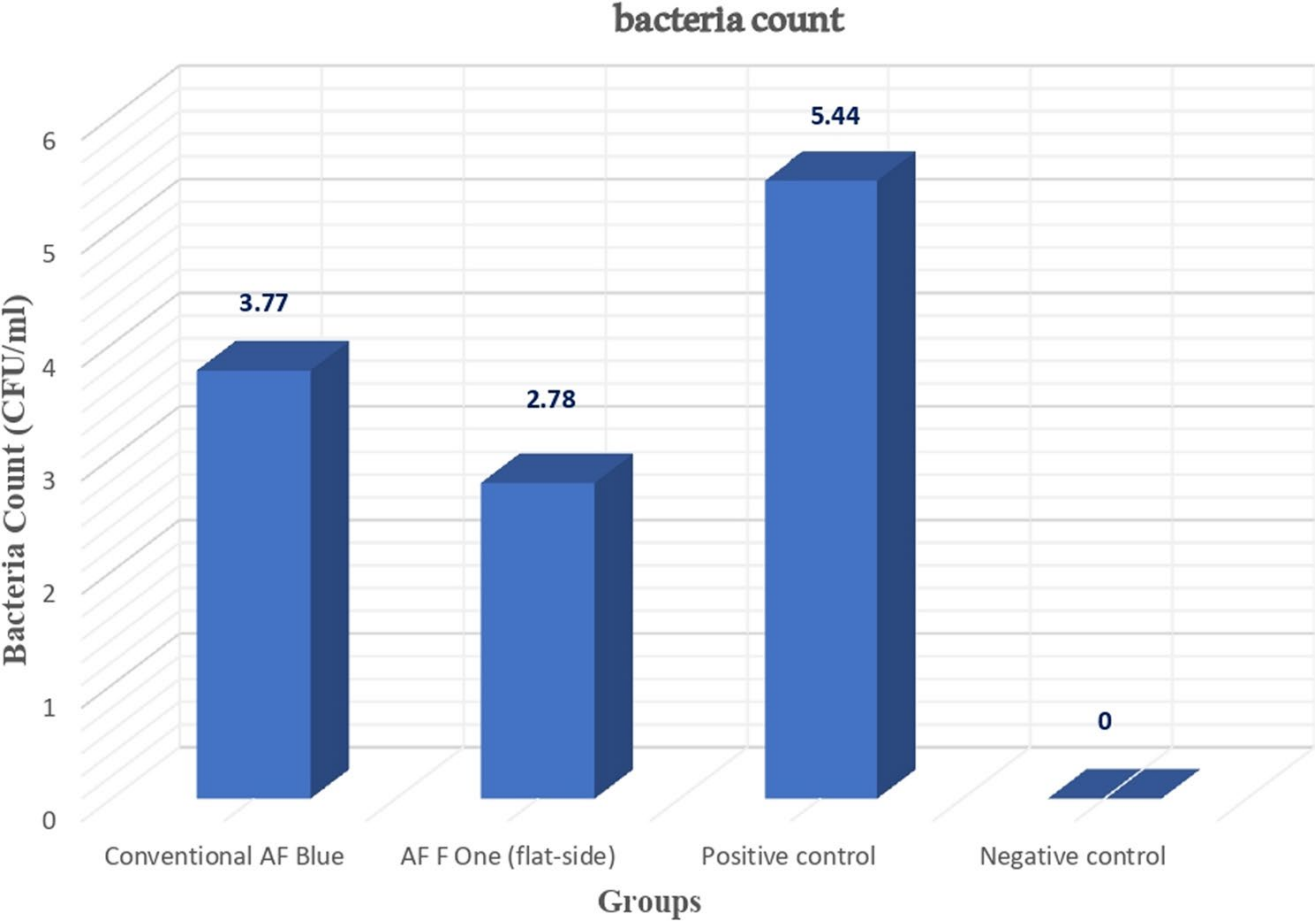
The mean log bacterial count (CFU/mL) for the different instrument designs and the control groups

Table 2 presents a comparison of the log bacterial count (CFU/ml) before instrumentation (S1) between the two experimental groups, ensuring consistent bacterial distribution (*p* = 0.94). It also provides a comparison of the log bacterial count (CFU/ml) for each instrument design between before and after instrumentation (S1 and S2). The mean and standard deviations of the log bacterial count after root canal preparation across the various study groups are summarized in Table 3 and illustrated in Fig. 1.

**Table 2:** A comparison of the log bacterial count (CFU/ml) before instrumentation (S1) between the two experimental groups, and a comparison for each instrument design between before and after instrumentation (S1 and S2). The results are presented as mean ± standard deviation

Groups	Bacterial count (CFU/ml)		*p*-value
	**S1 **	**S2**	
AF F One flat-side file system (FS)	5.43 ± 0.03	2.78 ± 0.11	0.006*
Conventional AF Blue file system (CP)	5.44 ± 0.03	3.77 ± 0.32	0.001*
	0.94	0.006*	

Statistical significance set at < 0.05

* Indicates statistical significance

**Table 3:** The mean and standard deviation of the log bacterial count (CFU/mL) after root Canal Preparation for the different instrument designs and the control groups

Groups	Bacterial count (CFU/mL) (means ± standard deviation)	A 95% confidence interval		*p-value*
		Lower limit	Upper limit	
Conventional AF Blue file system (CP)	3.77 ± 0.32^a^	3.54	4.00	0.00
AF F One flat-side file system (FS)	2.78 ± 0.11^b^	2.70	2.87	
Positive control (PC)	5.44 ± 0.02^c^	5.40	5.47	
Negative control (NC)	0.00 ± 0.00^d^	0.00	0.00	

Different superscript letters indicate significant differences

Significance was set at < 0.05

The findings indicate that while complete eradication of root canal infections was not achieved, the process of root canal preparation played a significant role in reducing the bacterial load present in the canals, regardless of the instrument design employed (*p* = 0.00).

When assessing the effectiveness of the different instrument designs in reducing bacterial burden, it was noted that flat-side instruments resulted in a markedly greater reduction in bacterial counts compared to their conventional counterparts. The mean bacterial count for flat-side instruments was 2.78 ± 0.11, with a 95% CI ranging from 2.70 to 2.87. In contrast, conventional classic designs had a mean bacterial count of 3.77 ± 0.32 and a 95% CI of 3.54 to 4.00. This difference was statistically significant (*p* < 0.05).

Furthermore, the results from the negative control group, which exhibited no signs of root canal infection (0.00 ± 0.00, with a 95% CI of 0.00 to 0.00), provided strong evidence supporting the sterility of the procedures conducted throughout the present experiment. Contrarily, the positive control group indicated the viability and growth of the inoculated bacteria throughout the experiment, presenting a mean bacterial count of 5.44 ± 0.02 and a 95% CI of 5.40 to 5.47.

## Discussion

Given the inherent limited effect of chemical antimicrobial agents on bacterial biofilm, mechanical instrumentation, irrespective of the type of mechanical instruments, is considered a core strategy to disrupt and reduce intra-radicular biofilm [8–11, 46].

One of the most recent advances in instrument design is the flat-side instruments. There is a lot of controversy in scientific literature surrounding the performance of flat-side designs in terms of their mechanical behavior, root canal debridement, and shaping abilities [23–31]. Still, the disinfecting capability of flat-side multi-file systems has not currently been evaluated, particularly since previous research found a direct correlation between the instrument design and the possibility of bacterial reduction from the root canal [11]. Therefore, the current preliminary study aimed to compare the influence of flat sides in rotary multi-file systems to their classic counterpart on root canal disinfection.

The current study was conducted on extracted teeth rather than using non-biological substrates, as it has been documented that dentin represents a favorable substrate for the adhesion and growth of the different microbial taxa [47]. It is worth mentioning that bovine teeth have been widely used in endodontic laboratory experiments because their physical characteristics and chemical composition closely resemble those of human teeth [48]. However, there are minor differences in the morphology of the dentin [48]. Therefore, extracted human teeth were selected to better simulate clinical situations. Due to their early eruption, human mandibular first molars are the most common tooth type vulnerable to carious lesions and frequently require root canal treatment even at young ages [49]. Furthermore, it has been demonstrated that effective root canal cleaning and disinfection of mandibular first molars would be complicated by the diverse anatomical variations of their root canal systems [50, 51]. Although this group of teeth has been associated with a variety of canal configurations [52], the mesial root has garnered increased attention due to its more intricate and variable anatomy, including the presence of inter-canal communications (isthmuses), a danger zone, extra canals, in addition to complex apical morphologies, which present challenges in cleaning and disinfection [53, 54]. Thus, the mesial root, with a Vertucci type IV canal configuration, of mandibular first molars was selected in the present study.

In this study, we opted to prepare access cavities instead of using decoronation, which has been employed in some previous microbiological investigations [36, 55]. This approach replicates the clinical scenario by creating an enclosed coronal space that allows for the retention and replenishment of irrigating solutions. Additionally, it can impact the subsequent root canal procedures [56, 57]. Traditional access cavities were prepared in the current experiment to ensure adequate access to the instruments without any interferences that could compromise their effectiveness, which may occur with minimally invasive access preparations [58–60].

In an important effort to consolidate the clinical relevance of this ex vivo investigation, unlike some microbiological studies that used saline or distilled water as the only root canal irrigants throughout their experiments [8, 11, 12], the present investigation employed the basic irrigation protocol used in routine clinical practice [37, 56]. In line with previous studies [36, 56], sodium thiosulfate was used to inactivate NaOCl.

AF F One is a heat-treated file system manufactured using the AF-R wire technique. According to the manufacturer, the AF-R wire can provide 600% higher cyclic fatigue resistance compared to the conventional NiTi alloy. This file system has a non-cutting tip that ensures safe progress of the file toward the apex, particularly in curved canals. The unique flat-side design featured in this system provides a space for storing irrigants inside the root canal during chemo-mechanical preparation, better debris removal in a coronal direction, apical advancement of the instrument with the least resistance, and more flexibility without negatively impacting the file’s strength.

*E. faecalis* is the most popular and tenacious bacterial species found in secondary and persistent endodontic infections and contributes to most of the persistent and/or failed cases [61–63]. This might be attributed to their ability to survive harsh environmental conditions, tolerate chemical disinfectants, and hide far away from the chemical agents and the mechanical action of instruments through deep penetration and colonization into inaccessible areas like dentinal tubules, isthmuses, apical ramifications, and accessory canals [64, 65]. Additionally, sampling and manipulation of *E. faecalis* can be simply and easily accomplished. It has been revealed that *E. faecalis* can survive in an ex vivo environment for an extended period of 12 months [64]. Research has indicated that the maturation timeline of each biofilm model is a crucial factor in determining the effectiveness of different disinfecting agents against biofilms [65]. The incubation period of 21 days has been found to increase the biofilm thickness, bacterial counts within the biofilm, and tolerance to antimicrobials without any discernible differences when the period has been extended [65]. Therefore, *E. faecalis* species were selected in the present investigation and incubated for 21 days.

The culture method using paper points is considered a simple and efficient approach to determining the bacterial burden and virulence factors [56, 66, 67]. It has been reported that the culture method can yield results similar to the molecular approaches [56]. Consequently, the culture method has been extensively implemented in numerous previous investigations [34, 55, 56, 68, 69]. To foster the internal validity of the obtained results, a small stainless steel H-file was used along all the canal walls in a pumping motion to aid in further disruption of the attached bacterial biofilms prior to sampling using paper points [8, 36, 68, 69].

Per the recent laboratory study guidelines (PRILE 2021), it is recommended to include both positive and negative control groups in microbiological investigations to assess the disinfecting abilities of the tested groups properly [70]. The positive control group is essential for assessing bacterial viability and growth throughout the experiment. It serves as a benchmark to confirm that the experimental conditions are conducive to bacterial proliferation and that the results can be interpreted accurately. In contrast, the negative control group plays an important role in ensuring that no bacterial contamination is introduced into the root canals due to the materials, solutions, and instruments used throughout the entire experiment. Therefore, both control groups were implemented in the current investigations.

In line with the compelling evidence [8–10, 12–15], the findings of the present study affirmed that total elimination of root canal infection is unattainable, and the root canal preparation, irrespective of the different instrument designs, was able to reduce the bacterial count significantly.

When comparing the disinfecting capabilities of different instrument designs, it was found that flat-side instruments significantly outperformed their standard counterparts in reducing the bacterial load within root canals. Consequently, the null hypothesis was rejected. The present findings agree with those of the Machado et al. study [11], suggesting that the instrument design may influence root canal disinfection.

Since both instruments tested in the present study are made by the same manufacturer and share similar characteristics except for a flat surface in the flat-side instruments, it was evident that the distinct flat side made a difference. The flat side might indeed create a reservoir for irrigants that could be activated while the file rotates inside the canal, enhancing chemical disinfection, along with the mechanical action of instruments. Another possible explanation for the superiority of the flat-side design over the conventional one is that the flat-side instruments may effectively remove infected debris, pulp remnants, and microorganisms from the active parts (flutes) to the relieving areas (flat surface) and transport them in a coronal direction toward the canal orifices beside the irrigation associated with the full-sequence rotary system employed in the present investigation, thereby improving root canal disinfection. This comes in contrast with the study by Silva et al. [26], which showed no significant difference in debris removal between the flat-side instruments and their classic counterparts. The discrepancy might be attributed to the different file systems and sequences, as well as the associated irrigation volumes and times. Additionally, the featured cutting efficiency of the flat-side instruments, as reported by Silva et al. [26], may help in the effective mechanical detachment of the bacterial biofilm from the canal walls.

The current findings did not align with the results of a previous study [32], which indicated that the flat-side instrument had a less effective disinfecting action than the conventional instruments. The inconsistency could be attributed to multiple differences in study designs, samples, file systems, and evaluation methods. The latter study compared file systems that differed in size, taper, alloy, manufacturing process, surface treatment, design, and the axis of rotation, making the comparison quite challenging. Additionally, it was conducted on mandibular single-rooted premolars with straight single oval canals prepared using a single-file system of a size of 25/0.06, which may be insufficient for disinfecting such wide and oval root canals, as supported by several studies [71–73]. Furthermore, the disinfecting capability in the latter study was assessed using a scanning electron microscope (SEM). Apart from the limited field of view, SEM is an invasive and destructive approach that can adversely impact the accuracy and reproducibility of the results [74].

The clinical relevance of the present study is underscored by its findings, which demonstrate that flat-side instruments may enhance disinfecting capabilities in root canal procedures. By improving bacterial reduction, these instruments have the potential to provide more effective treatment outcomes in clinical practice. This advancement is particularly crucial in minimizing the risk of persistent infections, which can complicate recovery and undermine the overall success of endodontic treatments. Enhanced disinfection practices can contribute to a thorough eradication of pathogens within the root canal system, ultimately promoting better healing and long-term success.

In addition to its status as a preliminary study with a limited sample size, the main constraint of this investigation appears to stem from its ex vivo nature. This design may impact the generalizability of the results, as the findings are based on observations conducted outside of a living organism. Consequently, the ex vivo conditions may not fully replicate the in vivo environment, potentially affecting the biological responses being measured. These factors collectively suggest that while the initial insights gained from this study are valuable, further research with larger samples and in more natural conditions will be necessary to draw more definitive conclusions. Nonetheless, the ex vivo environment provides favorable conditions to test the efficiency of new approaches, devices, instruments, and materials in a standardized manner, impossible to achieve in clinical studies. One of the strengths of the present study is that it followed the recent guidelines of laboratory investigations in endodontics in an effort to reach a vigorous design and precise execution and translate the obtained results into routine clinical practice [75]. Another significant strength of the current investigation resides in the innovative approach of integrating a flat-side design within a multi-file system. It was crucial to examine the influence of such a novel design on key factors, such as root canal disinfection, which are essential to successful root canal treatment. This aspect has been notably underexplored in the endodontic literature, making the research particularly valuable. By focusing on root canal disinfection—a critical component in preventing post-treatment complications—the study aimed to contribute fresh initial insights that could enhance clinical practices and outcomes in endodontics. This unique angle not only underscored the study’s originality but also highlighted its potential impact on improving the efficacy and safety of root canal procedures.

Due to the intricate and polymicrobial nature of root canal infections, one significant limitation of the present study is the use of single-species biofilms, which may not accurately represent the clinical situation typically encountered in practice. In real-world settings, root canal infections usually involve a diverse array of microorganisms that interact with one another, complicating the infection landscape. However, using monospecies biofilms offers certain advantages, such as enhanced standardization and better control, leading to greater reproducibility in laboratory studies when compared to multi-species biofilms [76, 77]. Integrating multiple bacterial species into biofilm models presents challenges, primarily due to the inconsistent distribution of bacteria across various study samples [77]. This inconsistency arises from variations in growth rates and environmental preferences among different bacterial taxa, which can affect their ability to co-exist and thrive in a mixed-species biofilm. Furthermore, the increased diversity and complexity inherent in multi-species biofilms can hamper the reproducibility of experimental results, complicating interpretations and comparisons [76, 77]. Considering these factors, single-species biofilms were selected in the current study. This approach allows for more precise control of experimental conditions and provides a clearer understanding of the behavior of individual bacterial strains.

Currently, there is no universally recognized gold standard for assessing the effectiveness of root canal disinfection [77, 78]. To address this issue, incorporating two or more complementary evaluation methods is suggested to offer a more comprehensive grasp of the disinfection process, particularly since each technique has its own pros and cons [78]. Therefore, future laboratory studies that utilize more than one approach to evaluate the disinfecting capability of such a novel design are necessary.

In summary, the present research highlighted the following points:

1. Total eradication of root canal infection is quite challenging.
2. Meticulous root canal instrumentation is a key strategy for reducing bacteria in root canals.
3. The design of instruments may influence the disinfection of root canals.
4. Compared to their traditional counterparts, flat-side multi-file rotary systems can reduce the bacterial load within the root canals.

While the current experiment adhered to the latest methodological standards in endodontic laboratory studies and yielded promising preliminary results, several limitations, along with future directions, must be acknowledged:

1. Limited sample size: Larger, well-powered investigations are necessary to validate these initial findings.
2. Biofilm variability: The present study utilized an in vitro monospecies biofilm model that may not fully represent the complex multispecies interactions encountered in clinical infections. Future research, including multispecies ex vivo or in vivo models, is important to establish a more translational approach.
3. Culture method limitations: Dependence on colony-forming unit (CFU) counting may underestimate microorganisms hidden in inaccessible locations inside the root canals, overlook viable but non-culturable (VBNC) bacteria, and does not evaluate residual biofilm structures. Using q-PCR, fluorescence-based viability assays, or advanced imaging techniques such as confocal laser scanning microscopy can allow for a more thorough assessment, particularly when applied in combination.
4. *Ex vivo* design: To enhance the validity of the present findings, further well-designed prospective randomized clinical trials are required to evaluate the clinical performance of the flat-side instruments and determine whether such a unique instrument design can affect the clinical and radiographic outcomes.

## Conclusion

The present preliminary laboratory investigation provides initial evidence supporting the potential capacity of the flat-side full-sequence rotary instruments to reduce the bacterial burden within root canals, particularly when compared to their classic counterparts. Further research with larger sample sizes and a range of multimodal biofilm assessment techniques is crucial to establish definitive conclusions.

**Fig. 2:**
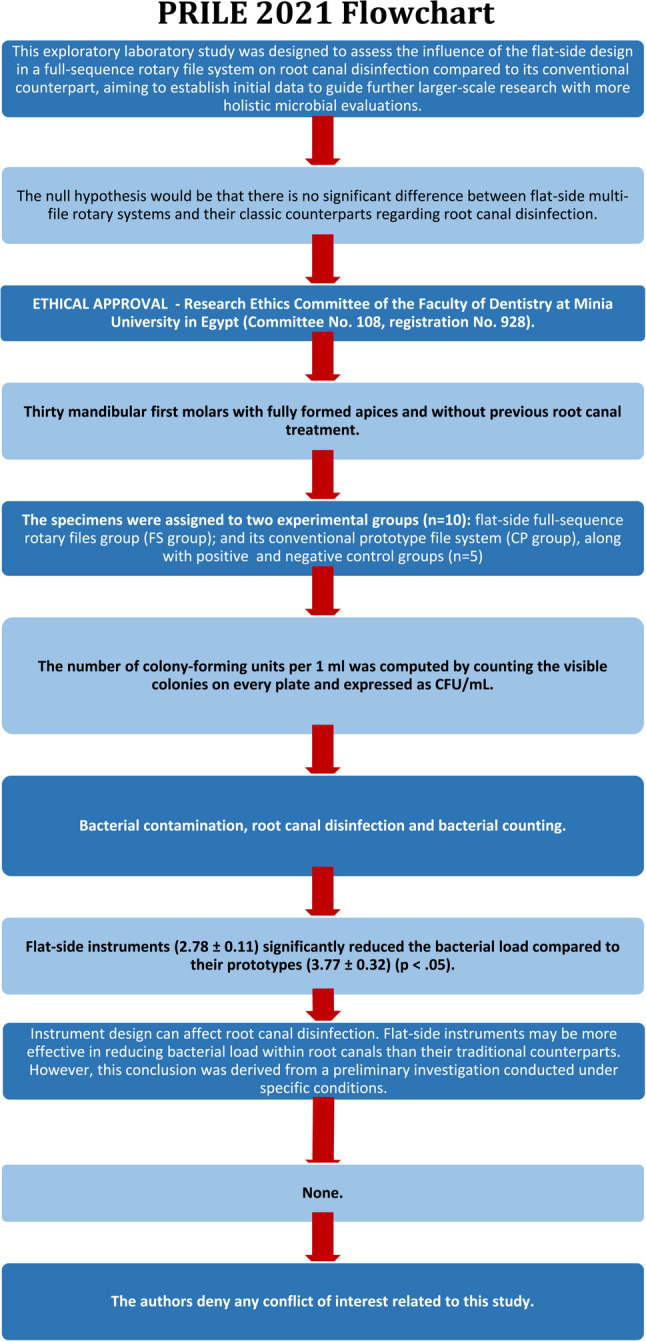
PRILE 2021 flowchart. *From Nagendrababu V, Murray PE, Ordinola-Zapata R, Peters OA, Rôças IN, Siqueira JF Jr, Priya E, Jayaraman J, Pulikkotil SJ, Camilleri J, Boutsioukis C, Rossi-Fedele G, Dummer PMH (2021) PRILE 2021 guidelines for reporting laboratory studies in Endodontology: a consensus-based development. International Endodontic Journal May 3. 10.1111/iej.13542. https://onlinelibrary.wiley.com/doi/abs/10.1111/iej.13542. For further details visit: http://pride-endodonticguidelines.org/prile

This manuscript has been meticulously structured according to the Preferred Reporting Items for Laboratory studies in Endodontology (PRILE 2021) guidelines [70]. These established standards provide a comprehensive framework that ensures transparency, consistency, and rigor in reporting laboratory studies specifically related to the field of endodontics. The findings of the present preliminary laboratory study are concisely summarized in the PRILE 2021 flowchart, which is provided as Fig. 2. This flowchart serves as a visual aid, representing the pivotal components of the study, including the primary objective, the null hypothesis established at the outset, the methodology employed for data collection and analysis, and a summary of the results obtained. Furthermore, it outlines the conclusions drawn from the data, thereby enhancing the reader’s comprehension of the research process and its outcomes. By clearly delineating these elements, the flowchart acts as a valuable tool for readers to grasp the essential aspects of the study and its implications within the broader context of endodontic research.

## Supplementary Information

Supplementary Material 1.

## Data Availability

All data or materials generated or analyzed during this study are included in this article.

## Abbreviations

NaOCl: Sodium hypochlorite
E. faecalis: Enterococcus faecalis
ATCC: American Type Culture Collection
BHI: Brain-heart infusion
CFU: Colony-forming unit
nm: Nanometer
CI: Confidence interval
PRILE: Preferred Reporting Items for Laboratory Studies in Endodontology
ANOVA: Analysis of variance
ISO: International Organization for Standardization
EDTA: Ethylenediaminetetraacetic acid
PC: Positive control
NC: Negative control
^o^ C: Celsius
h: Hours
min: minutes
S: Sampling
s: Seconds
mm: Millimeter
mL: Milliliter
